# Advances in the Surgical Management of Cervical Cancer

**DOI:** 10.3390/cancers18040628

**Published:** 2026-02-14

**Authors:** Lakeisha Mulugeta-Gordon, Minyoung Jang, Christian Dagher, Dimitrios Nasioudis

**Affiliations:** 1 Division of Gynecologic Oncology, Department of Obstetrics and Gynecology, Perelman School of Medicine, University of Pennsylvania, Philadelphia, PA 19104, USA; lakeisha.mulugeta-gordon@pennmedicine.upenn.edu; 2 Department of Obstetrics and Gynecology, Perelman School of Medicine, University of Pennsylvania, Philadelphia, PA 19104, USA; minyoung.jang@pennmedicine.upenn.edu (M.J.); christian.dagher@pennmedicine.upenn.edu (C.D.)

**Keywords:** cervical cancer, surgical procedures, surgical staging, fertility sparing surgery, minimally invasive surgical approaches, cervical cancer oncologic outcomes

## Abstract

Cervical cancer is the third most common gynecologic malignancy in the United States. Traditionally, a radical hysterectomy with bilateral salpingo-oophorectomy with lymph node assessment had been the standard of care; however, there has been a recent shift towards less radical surgery with the establishment of the oncologic safety of conization (as fertility preserving surgery), and simple hysterectomy for certain subgroups of patients with early-stage disease. In addition, uptake in the utilization of sentinel lymph node mapping, compared to systematic lymphadenectomy, has translated to improved perioperative outcomes. While laparotomy is the preferred mode of surgery for patients with cervical cancer, emerging data suggest that protective maneuvers may not compromise oncologist outcomes and multiple clinical trials are evaluating the oncologic safety of minimally invasive surgery. In this present review, we summarize recent advances in surgical management in patients with cervical cancer.

## Simple Summary

This review illustrates the evolving history and the emerging landscape of surgical management in cervical cancer. Historically, standard surgical practice included a radical hysterectomy and lymphadenectomy via a laparotomy incision. Over the years, the indications for radical surgery have decreased given the increased surgical morbidity and worse quality of life. This review will highlight the innovative surgical history in cervical cancer including when a simple hysterectomy is appropriate, the consideration for minimally invasive surgery, sentinel lymph node mapping and excision, fertility-sparing when appropriate, and staging in locally advanced and pelvic exenteration, while reviewing oncologic outcomes and patient-reported outcomes. Further, this review will highlight ongoing trials.

## 1. Introduction

Cervical cancer is the third most common gynecologic cancer in the United States, with roughly 13,000 new cases and 4000 deaths annually [1]. In the United States, cervical is the fourteenth most common cancer and it is the fourth for women aged 15–44 years [2]. Despite immunizations and cervical cancer screening with cervical cytology and human papilloma virus genotyping, cervical cancer remains a public health concern [3]. This concern is more significant worldwide, where it is the fourth most common cancer in the world, primarily affecting women in low-resource countries [3]. A recently published study by Sathiyaseelan et al. conducted a systematic review assessing sociocultural factors’ influence on cancer screening for multiple cancer types including cervix. Using the Health Belief Model and Socioeconomic model, the authors found that there are multiple individual- and cultural-level considerations that must be factored in when educating patients on the importance on cancer screening [4]. Thus, adherence to guideline concordant care for cervical cancer very much depends on access to such care. Cervical cancer recommendations depend on the stage at presentation with options ranging from surgery to radiation, chemoradiation, and systemic therapy.

The management of cervical cancer differs from the two more common gynecologic cancers, endometrial and ovarian, in that surgical management is not recommended for locally advanced or advanced disease unless it is for curative intent. Traditionally, for early-stage cervical cancer, a radical hysterectomy with full pelvic lymph node assessment was the standard of care followed by observation or either adjuvant radiation or radiotherapy depending on if the patient met Sedlis or Peters criteria, respectively [5,6]. However, radical hysterectomy with full pelvic lymph node assessment carries a high cure rate but is not a surgical procedure without risk. This review article dives into the key aspects of surgery for cervical cancer including when to consider a simple versus radical hysterectomy for early-stage disease as well as the optimal hysterectomy approach: laparotomy versus minimally invasive. In addition, we summarize the literature behind sentinel lymph node mapping and excision in cervical cancer, which patients are candidates for fertility-sparing options, and the controversial debate over staging in locally advanced cervical cancer. A summary of key completed surgical trials in cervical cancer can be found in Table 1. Ongoing trials in cervical cancer can be found in Table 2. Lastly, we will review pelvic exenterations as an option for patients with a central recurrence, but a procedure that is life-changing and requires precise patient selection. Our literature review utilized the Cochrane, Embase, and PubMed databases from July to December 2025 to write this review.

**Table 1:** Key prospective and randomized controlled trials in surgical management of cervical cancer.

Trial, Author (Publication Year)	Trial Design	Study Aim/Primary Endpoint	Inclusion Criteria	Patient Randomization	Key Findings	Conclusion
**Simple vs. Radical surgery**						
LESSER, Carniero et al. (2022) [7]	Phase II, multicenter, randomized, non-inferiority	To evaluate the non-inferiority and safety of simple hysterectomy in early-stage cervical cancer Primary endpoint: 3-year disease-free survival (DFS)	FIGO 2009 IA2-IB1, tumor size ≤ 2 cm, adenocarcinoma, squamous, or adenosquamous, all histologic grades	• Simple hysterectomy (n = 20) • Type B modified radical hysterectomy (n = 20) • All patients underwent a pelvic lymphadenectomy	3-year DFS: 95% for simple vs. 100% for radical (*p* = 0.30) 5-year OS was 90% for simple vs. 91% for radical (*p* = 0.46)	Simple hysterectomy is safe and potentially non-inferior to the radical surgery in patients with early-stage cervical cancer ≤ 2 cm
SHAPE, Plante et al. (2024) [8]	Phase III, multicenter, randomized, non-inferiority	To evaluate the safety of simple hysterectomy compared with radical hysterectomy in patients with low-risk early-stage cervical cancer Primary endpoint: 3-year pelvic recurrence	FIGO 2009 IA2-IB1, adenocarcinoma, squamous, or adenosquamous, low risk features (≤2cm, all grades, limited stromal invasion, <10 mm on LEEP or CKC or <50% on MRI, no nodal mets); presence of LVSI was not excluded	• Simple hysterectomy (n = 350) • Type B modified radical hysterectomy (n = 350) • All patients underwent a pelvic lymphadenectomy or sentinel lymph node biopsy	3-year pelvic recurrence: 2.52% (simple) vs. 2.17% (radical) Urinary retention within 4 weeks of surgery: 0.6% (simple) vs. 11% (radical) Urinary incontinence: 2.4% (simple) vs. 5.5% (radical) No significant difference in pelvic recurrence-free survival, extra-pelvic recurrence-free survival, overall recurrence-free survival, or overall survival	Simple hysterectomy was not inferior to radical hysterectomy and was associated with a lower risk of urinary incontinence or retention
GOG 0278, Covens et al. (2025) [9]	Prospective, international	To assess patient outcomes before and after cone biopsy (CB) or simple hysterectomy (SH) with pelvic lymph node dissection (PLND)	FIGO 2018 IA1 (+LVSI)-IB1 (≤2 cm; adenocarcinoma, squamous, or adenosquamous, all histologic grades	• Cone biopsy (N = 72) • Simple hysterectomy (N = 152) • All patients underwent a pelvic lymphadenectomy • Not randomized	There was a temporary decline in bladder, bowel, and sexual function postop, with gradual recovery. QOL improved and cancer-related worry decreased over time. Lymphedema was reported by 12 patients (6 in each group)	Cone biopsy or simple hysterectomy is associated with improved quality of life and small decline in sexual, bladder, and bowel functions
**Minimally invasive surgery vs. Laparotomy**						
LACC, Ramirez (2018) [10]	Phase III, multicenter, randomized, non-inferiority	Compare disease-free survival between minimally invasive and open radical hysterectomy Primary endpoint: 4.5-year disease-free survival	Stage IA1 (+LVSI)-IB1 (≤4 cm); adenocarcinoma, squamous, or adenosquamous	• Minimally invasive (N = 319) • Open (N = 312) • All patients underwent a pelvic lymphadenectomy	3-year DFS: 91.2% (MIS) vs. 97.1% (open) 3-year OS: 93.8% (MIS) vs. 99.0% (open) Results confirmed in final analysis published in 2024	MIS was associated with lower rates of disease-free survival and overall survival than open abdominal radical hysterectomy among women with early-stage cervical cancer
**Sentinel lymph node mapping**						
SENTICOL, Lecuru et al. (2011) [11]	Prospective,	To assess if sentinel lymph node biopsy is acceptable in surgical staging for early-stage cervical cancer	Stage IA1 (+LVSI)-IB1 (≤4 cm); adenocarcinoma, squamous, or adenosquamous	Not randomized	Out of 136 patients, there were 2 false negatives Sensitivity: 92% Negative predictive value: 98.2%	Combined labeling for node mapping was associated with high rates of SLN detection and with high sensitivity and NPV for metastasis detection
SENTIX, Cibula et al. (2025) [12]	Prospective, Phase III, multicenter, non-inferiority	To assess if sentinel lymph node biopsy is acceptable in surgical staging for early-stage cervical cancer Primary endpoint: recurrence rate	Stage IA1 (+LVSI)-IB2; adenocarcinoma, squamous, or adenosquamous	Not randomized	Bilateral detection in 91% Rate of lymph node metastasis: 12% Ultrastaging identified 44% of lymph node mets	Sentinel lymph node biopsy is feasible in tumors ≤ 4 cm
PHENIX I, Hua et al. (2025) [13]	Multicenter, non-inferiority, randomized	To compare survival outcomes between sentinel lymph node and pelvic lymphadenectomy (after sentinel lymph node) Primary endpoint: disease-free survival	Stage IA1 (+LVSI)-IB1, IIA1 adenocarcinoma, squamous, or adenosquamous Intraoperative randomization	• Total patients: N = 908 • SLN negative (N= 838) • SLN positive (N = 70)	Bilateral detection in 82.6% 3-year DFS: 96.8% (SLN) vs. 94.5% (PL)—not statistically significant	If sentinel lymph node maps, it is safe to omit a pelvic lymphadenectomy
**Fertility sparing**						
ConCerv, Schmeler et al. (2021) [14]	Prospective, multicenter	To evaluate the feasibility of conservative surgery in women with early-stage, low-risk cervical cancer	FIGO 2009 Stage IA2-IB1; Squamous (any grade), adenocarcinoma (grade 1 or 2) No LVSI, needed cone biopsy to be enrolled	• Cone biopsy followed by lymph node assessment (N = 44) • Cone biopsy followed by simple hysterectomy and lymph node assessment (N = 40) • Simple hysterectomy followed by lymph node assessment	Recurrence rate for patients with cone biopsy: 1/44 Recurrent rate for patients with cone biopsy followed by simple hyst: 0/36 Recurrence rate for patients with simple hyst: 2/16	Select patients can be offered fertility-sparing option for early-stage cervical cancer management
**Surgical staging in locally advanced cervical cancer**						
LiLACS, Frumovitz et al. (2014) [15]	Phase III, multicenter, randomized	To compare survival outcomes between pre-therapeutic laparoscopic surgical staging followed by tailored chemoradiation and radiologic staging alone followed by chemoradiation in locally advanced cervical cancer Primary endpoint: overall survival	Stage IB2-IVA, adenocarcinoma, squamous, or adenosquamous histologies Lymph node criteria: (1) FDG-positive or indeterminate pelvic lymph nodes, (2) FDG-negative para-aortic nodes	Not applicable	Not applicable	Closed due to poor accrual
UTERUS-11, Marnitz et al. (2020) [16]	Phase III, international multicenter, randomized	To compare disease-free survival outcomes between surgical staging and radiologic staging followed by chemoradiation in locally advanced cervical cancer Primary endpoint: disease-free survival	FIGO 2009 Stage IIB-IVA adenocarcinoma, squamous, or adenosquamous histologies	• Surgical arm (N = 130) • Clinical arm (N = 125)	No difference in disease-free survival between both arms 98% of patients had adjuvant external beam radiation	

**Table 2:** Key ongoing prospective and randomized controlled trials in surgical management of cervical cancer.

Trial, Author (Publication Year)	Study Design	Study AIM	Primary Endpoint	Inclusion Criteria	Target Participants
**Simple vs. Radical surgery**					
LASH, Bizarri et al. (2025) [17]	Single-arm, prospective	To assess the oncologic outcomes for patients with early-stage cervical cancer that undergo a minimally invasive hysterectomy	3-year disease-free survival	FIGO 2018 Stage IA2-IB1, ≤10 mm DOI, ≤50% invasion on pre-conization MRI; squamous, HPV-associated adenocarcinoma, and adenosquamous histologies	974
**Minimally invasive surgery vs. Laparotomy**					
RACC, Falconer et al. (2019) [18]	International, multicenter, open label randomized clinical trial; 1:1 randomization, non-inferiority	To assess and compare oncologic outcomes for patients with early-stage cervical cancer that undergo a minimally invasive radical hysterectomy and open radical hysterectomy	5-year recurrence-free survival	FIGO 2018 Stage IB1, IB2, IIA1; squamous, adenocarcinoma, and adenosquamous histologies	768
ROCC, Leitao et al. (2025) [19]	Multicenter, open label randomized clinical trial; 1:1 randomization, non-inferiority	To assess and compare oncologic outcomes for patients with early-stage cervical cancer that undergo a minimally invasive hysterectomy and open radical hysterectomy (simple and radical approaches included)	3-year disease-free survival	FIGO 2018 Stage IA2-IB2; squamous, adenocarcinoma, and adenosquamous histologies. Tumor less than 4 cm on preoperative MRI	840
**Sentinel lymph node mapping**					
SENTICOL-III, Lecuru et al. (2019) [20]	International, multicenter, randomized, single-blinded	To assess and compare oncologic outcomes for patients with early-stage cervical cancer that undergo sentinel lymph node biopsy only and sentinel lymph node biopsy, followed by pelvic lymphadenectomy	3-year disease-free survival and health-related quality of life	FIGO 2018 Stage IA1 with LVSI-Stage IIA, ≤4 cm; squamous and adenocarcinoma histologies	950
**Fertility sparing**					
CONTESSA/NEOCON-F, Plante et al. (2019) [21]	International, multicenter, prospective	To evaluate the feasibility of preserving fertility	Assess the rate of functional uterus defined as successful fertility-sparing surgery and no adjuvant therapy	FIGO 2018, Stage IB2 (2–4 cm), pre-menopausal (≤40 years old)	Expected to enroll 90 by end of 2025
FERTISS Study, Fricová et al. (2024) [22]	International multicenter retrospective observational study	Analyze oncological outcomes and reproductive outcomes after fertility-sparing treatment	Recurrence rate and reproductive outcomes (conception attempts, pregnancy success, delivery outcomes)	Age 18–40 years; FIGO 2018 stage IA1 with positive LVSI or ≥IA2; any type of fertility-sparing procedure; regardless of histotype, tumor grade, or neoadjuvant chemotherapy history	733
**Surgical staging in locally advanced cervical cancer**					
PAROLA, Martinez et al. (2023) [23]	International, multicenter, randomized, Phase III; 1:1 randomization	To compare oncologic outcomes in patients who receive chemoradiation tailored by surgical staging and pathologic review of para-aortic lymph nodes and patients staged with imaging alone (PET/CT)	3-year disease-free survival	Histologically proven Stage IIIC1 cervical cancer	510
**Pelvic exenteration in recurrent cervical cancer**					
MIPEX, Bizzarri et al. (2025) [24]	Single-arm, interventional, non-randomized study (single group assignment, no masking)	To assess oncologic safety of minimally invasive pelvic exenteration in recurrent/persistent cervical or vaginal cancer suitable for PE per ESGO guidelines	3-year disease-free survival (DFS), measured from enrollment for 3 years	Recurrence or persistence of cervical or vaginal cancer with pelvic location	64

## 2. Simple vs. Radical Surgery for Early-Stage Disease

There is a growing movement toward surgical de-escalation for patients with low-risk early-stage cervical cancer. Historically, radical hysterectomy with pelvic lymphadenectomy was the standard of care for patients with early-stage disease. However, radical surgery carries significant risks of perioperative complications and morbidity related to the extent of parametrial resection, namely the effects on the bladder, bowel, and sexual functions [25,26]. Numerous retrospective studies have demonstrated that less than 1% of early-stage cervical cancers with low-risk pathological features (tumor size < 2 cm, depth of invasion < 10 mm, no nodal disease) exhibit parametrial involvement, suggesting that less radical surgical approaches may offer equivalent oncologic outcomes with reduced morbidity [27,28,29,30,31]. The quality of data examining this topic had been modest until several recent paradigm-shifting studies [7,32]. An early proof-of-concept study, the LESs Surgical Radicality for EaRly Stage Cervical Cancer (LESSER) trial (2023), was a Phase 2, multicenter, randomized, non-inferiority trial evaluating the efficacy and safety of conservative surgical management for early-stage cervical cancer [7]. Patients with FIGO 2009 stage IA2 to IB1 cervical adenocarcinoma, squamous, or adenosquamous cancer and tumors ≤ 2 cm were included. Notably, the depth of invasion, histological grade 3, and presence of lymphovascular space invasion (LVSI) were not considered exclusion criteria. Half of the patients had a simple hysterectomy (type A, n = 20) and the other half had a modified radical hysterectomy (type B, n = 20). In addition to the hysterectomy, all patients had a pelvic lymph node dissection. The 3-year disease-free survival rate was 95% (95% CI: 68% to 99%) after simple hysterectomy and 100% (95% CI: 100% to 100%) after modified radical hysterectomy, with a *p*-value of 0.30. Rates of 5-year overall survival were 90% (95% CI: 64% to 97%) vs. 91% (95% CI: 50% to 98%), respectively, with a *p*-value 0.46. Patients who had a simple hysterectomy had shorter operative times and a shorter time to removal of the urinary catheter. This trial was a key early trial in supporting simple hysterectomies for patients with early-stage cervical cancer.

In this context, the Simple Hysterectomy and Pelvic Nodal Assessment (SHAPE) trial (2024) was published, the most robust and practice-changing study to date. This was a landmark Phase 3, multicenter, randomized, non-inferiority trial comparing cancer recurrence between radical and simple hysterectomy [8]. Patients with FIGO 2009 stage IA2 to IB1 cervical cancer and the following low-risk features were included: squamous cell carcinoma, adenocarcinoma, or adenosquamous carcinoma, lesion size ≤ 2 cm, any histologic grade, limited stromal invasion (<10 mm on LEEP or cone biopsy or <50% on preoperative MRI), and no evidence of nodal metastasis. Presence of LVSI was not an exclusion criterion. A total of 700 patients were assigned to type II radical hysterectomy (n = 350) or simple hysterectomy arms (n = 350). With a median follow-up of 4.5 years, the incidence of pelvic recurrence at 3 years was 2.52% in those who underwent simple hysterectomy and 2.17% in those who underwent radical hysterectomy (absolute difference 0.35%, 90% CI: 1.62, 2.32). Moreover, there were no significant differences in pelvic recurrence-free survival, extra-pelvic recurrence-free survival, recurrence-free survival, or overall survival between groups. Of note, 0.6% of patients in the radical hysterectomy group had distant recurrences as opposed to 2% in the simple hysterectomy group. Subgroup analyses did not reveal differences by histology, LVSI status, or surgical approach, though the trial included only a small proportion of patients with grade 3 adenocarcinoma or LVSI. Fewer urinary complications, particularly retention and incontinence, as well as better quality of life and short-term and long-term sexual health outcomes were associated with simple hysterectomy [33]. The SHAPE trial redefined the standard of care for early-stage low-risk cervical cancer, demonstrating that conservative approaches can maintain oncologic outcomes while reducing surgical morbidity and preserving quality of life. Criticisms of the SHAPE trial include the choice of primary endpoint (3-year pelvic recurrence rate), as well as the high utilization of minimally invasive techniques (~70% and ~80% in the radical and simple hysterectomy groups, respectively) and possible surgical quality issues (given the presence of positive vaginal margins in ~3% of patients randomized to the radical hysterectomy arm).

An important consideration of oncologic outcomes, primarily the risks of recurrence or relapse, in early-stage cervical cancer is conization prior to radical hysterectomy. The goal of a cold knife cone or cone biopsy is to evaluate dysplasia or carcinoma with the goal of obtaining negative margins. For cone biopsies that show carcinoma with negative margins and no LVSI, observation if the patient desires fertility or a simple hysterectomy can offer if the patient meets SHAPE criteria. For patients where a radical hysterectomy is indicated, their risk of relapse may be lower if they had a prior cone. Two epidemiologic studies sought to answer this question. Chacon et al. conducted a multicenter, retrospective study, SUCCOR, in patients with FIGO 2009 Stage IB1 cervical cancer over a 2-year period. They used 1:1 propensity score matching and compared patients who had a cone biopsy to patients who did not have a cone biopsy [34]. Their cohort included 374 patients, 187 patients with a prior cone & and 187 without a prior cone. They found that patients who had a prior cone had 65% lower risk of recurrence (HR 0.35, 95% CI: (0.16, 0.75)) and 75% lower risk of death (HR 0.25, 95% CI: (0.70, 0.90)) [34]. Furthermore, for patients who did not have a prior cone and had a minimally invasive hysterectomy, their risk of recurrence was 5.64 times higher than patients who had a prior cone and a radical hysterectomy via laparotomy [34]. There were no statistically significant differences between patients who had prior cone and minimally invasive hysterectomy and patients who did not have a prior cone and had a laparotomy [34]. These findings are consistent with another retrospective conducted by Nasioudis et al., which also assessed oncologic outcomes in patients with FIGO 2009 Stage IB1cervical cancer who had a prior cone. This study utilized the national cancer database and included 3159 patients [35]. One third of the patients in this cohort underwent a cervical excisional procedure. This study found that patients with a prior excisional procedure had better overall survival after controlling for confounders compared to patients who did not [35]. Furthermore, patients with a prior cone had lower rates of lymphovascular invasion, positive lymph nodes, and tumor size < 2 cm, which were all statistically significant [35]. Outcomes were not worse for patients who had a minimally invasive hysterectomy, if they had a prior cone. Although retrospective, these data illustrate that prior cervical excisional procedures may confer superior oncologic outcomes in patients who subsequently undergo a radical hysterectomy for early-stage cervical cancer. Caution should be exercised when interpreting these data given the inconsistent measurement of total tumor size and possible patient selection bias since those with smaller, biologically less aggressive tumors are more likely to undergo conization.

Most recently, the GOG-0278 trial (2025) presented data from a prospective, international study of patients with FIGO 2018 stage IA1 (LVSI positive) to IB1 (≤2 cm) cervical cancer stratified by fertility-sparing cone biopsy (n = 72) or non-fertility-sparing simple hysterectomy (n = 152), both with pelvic lymphadenectomy [9,36]. Additional inclusion criteria were ≤10 mm stromal invasion and negative margins on LEEP or cone biopsy, in contrast with the SHAPE criteria which permitted the assessment of stromal invasion on preoperative imaging. Less radical surgery was associated with excellent quality of life, low rates of lymphedema, and overall minimal impact on bladder, bowel, and sexual functions with return to baseline postoperatively in both groups [36]. Secondary aims of this study included assessing recurrence, morbidity, and reproductive outcomes [9]. Recurrence-free survival was 94.8% (95% CI 89% to 100%) at a median follow-up of 37 months. Three recurrences occurred in the cone biopsy group alone; all were confined to the cervix and successfully treated with subsequent surgery. Interestingly, all patients with recurrence had negative margins on their cone biopsy and ECC, underscoring the importance of regular cervical assessments during surveillance post-surgery. One patient who underwent cone biopsy and seven patients who underwent simple hysterectomy experienced grade ≥ 3 adverse events within 30 days of surgery. With regard to reproductive outcomes in the cone biopsy group, 16 pregnancies occurred during the study period. Of these, there were four (25%) spontaneous abortions, three (19%) preterm deliveries, and nine (56%) full-term deliveries. In terms of the mode of delivery, seven (58%) had vaginal deliveries, and five (42%) had cesarean deliveries. The findings from GOG-0278 further support the safety and improved quality of life with less radical surgery for early-stage low-risk cervical cancer, especially for patients who may desire fertility-sparing surgery.

An area of ongoing research is the route of simple hysterectomy, as none of the studies assessing radical vs. conservative surgical approaches were designed to compare the safety of minimally invasive surgery (MIS) versus laparotomy. Presently underway, the LASH trial is a prospective, single-arm study aimed at evaluating the safety and feasibility of MIS simple hysterectomy for low-risk cervical cancer using SHAPE criteria [17].

## 3. Minimally Invasive Surgery vs. Laparotomy

For patients with early-stage cervical cancer, who are not candidates for fertility-sparing or simple hysterectomy and are surgical candidates, the standard of care surgical recommendation is for a radical hysterectomy with bilateral salpingectomy vs. salpingo-oophorectomy depending on the patient’s age and discussion regarding ovarian preservation. There have been multiple studies looking at cancer-specific outcomes between open laparotomy and the minimally invasive approach.

Originally, retrospective and observational data published by multiple groups had demonstrated similar oncologic outcomes between open surgery and conventional laparoscopy [37,38]. A systematic review and meta-analysis by Shazly et al. reported that the MIS approach is equivalent to the abdominal approach and may even be superior in terms of intraoperative and perioperative outcomes [39].

In 2017, Rameriz et al. published the Phase III LACC trial, which changed the practice of the surgical management of early-stage cervical cancer. The aim of the LACC trial, which was designed as a non-inferiority trial, was to compare MIS radical hysterectomy (either robotic or laparoscopic) to abdominal radical hysterectomy in patients with IA1 with LVSI, IA2, or IB1 squamous, adenocarcinoma, or adenosquamous cervical cancer [40]. Their results, which were reported at 3 years, demonstrated that patients who underwent a hysterectomy via an MIS approach had a lower disease-free survival, 91.2% vs. 97.1% (HR 3.74, 95% CI: 1.63, 8.48), which was their primary endpoint. Overall survival was a secondary endpoint, which also demonstrated that patients who had an MIS procedure had a lower survival rate, 93.8% vs. 99.0% (HR 6.00, 95% CI: 1.77, 2.30). The final analysis of the LACC trial published in 2024 confirmed these findings, with the final conclusion that radical abdominal hysterectomy remains the standard of care for early-stage cervical cancer [10]. Postoperative quality of life was comparable between the two groups with differences in mean FACT-Cx scores 6 weeks and 3 months post-surgery. Criticisms of the study include lack of vaginal protective maneuvers, variation in practice across participating sites, some of which had lower surgical volume, and the high utilization of traditional laparoscopy as opposed to robotic surgery.

Prior to the publication of the LACC trial, hysterectomy via minimally invasive surgery was an acceptable approach for patients with early-stage cervical cancer, without a robust amount of evidence regarding its safety. Two epidemiologic studies assessed survival in patients with early-stage cervical cancer who underwent minimally invasive hysterectomy. In a cohort study utilizing propensity score matching by Melamed et al., patients who underwent a minimally invasive radical hysterectomy (primarily via robotic approach) had a shorter overall survival, with the risk of death at 4 years being 9.1% in the minimally invasive group and 5.3% in the laparotomy group [41]. Furthermore, in 2020, Nitecki et al. published a systematic review and meta-analysis to assess whether the current literature was robust enough to support that minimally invasive surgery for early-stage cervical (FIGO IA1-IIA) cancer was associated with worse survival and higher risk recurrence [42]. This study included 15 studies with a total of 9419 patients. Patients who underwent a minimally invasive hysterectomy had a 70% higher risk of having recurrence compared to laparotomy [42]. The risk of death was 40% higher in the minimally invasive surgery group [42]. However, what about for patients with stage IA disease? A study by Nasioudis et al. assessed oncologic outcomes for patients with stage IA cervical cancer who under a minimally invasive hysterectomy. The majority of the patients in this study were stage IA1 (73.3%) and just half were treated with a robotic hysterectomy (41.8%) [43]. This study did not demonstrate worse oncologic outcomes with a minimally invasive approach. An exploratory analysis from the SHAPE trial supports this finding. In this exploratory analysis, 281 (83%) of patients underwent a robotic hysterectomy [44]. Twelve patients (4.3%) and three (5.3%) in the minimally invasive and the laparotomy groups, respectively, had a recurrence by 4.5 years [44]. Additionally, there were no differences in oncologic outcomes. It is important to note that with the increased utilization of robotic surgery, further trials are indicated. The LASH trial (assessing minimally invasive simple hysterectomy in low-risk cervical cancer), the RACC trial (assessing robot-assisted surgery vs. laparotomy for early-stage cervical cancer in Europe), and the ROCC trial (assessing robot-assisted surgery vs. laparotomy for early-stage cervical cancer in the United States) are ongoing surgical trials for the management of early-stage cervical cancer. RACC is closed for accrual, while ROCC and LASH are enrolling patients [17,18,19]. These trials prohibit the use of intra-uterine manipulators and implement protective maneuvers to minimize intraperitoneal tumor exposure, while, in an attempt to minimize poor surgical technique, strict surgeon selection criteria for participation are applied. These non-inferiority randomized trials may once again alter the current recommendations.

## 4. Sentinel Lymph Node Mapping for Cervical Cancer

Surgical evaluation of lymphatic basins is recommended for patients with apparent early-stage disease since the presence of lymph node metastases can guide adjuvant treatment recommendations. Traditionally systematic bilateral pelvic lymphadenectomy was performed with increased perioperative morbidity and rates of postoperative lymphedema [45]. The SENTICOL trial first prospectively evaluated whether sentinel lymph node biopsy (SLNBx) alone is acceptable in the surgical staging of patients with early cervical cancer. In the intention to treat a population that included 136 patients, only two false negative results were observed with an overall 92% sensitivity and 98.2% negative predictive value [11]. The SENTIX trial was a prospective non-inferiority trial designed to evaluate the safety of SLNBx in patients with apparent early-stage cervical cancer [12]. It enrolled a total of 731 patients between 2016 and 2020 with FIGO stage IA1 (with LVSI)–IB2 disease. Based on 594 patients who underwent SLNBx alone without systematic lymphadenectomy, the overall rate of lymph node metastases was 12% (82 patients) while at two years the rate of disease relapse was 6.1% with a 2-year overall survival rate of 97.9%. Of note, ultrastaging identified almost half of lymph node metastases (44%) [12]. Another multicenter prospective randomized trial enrolled patients between 2015 and 2023 with FIGO 2009 stage IA1 (with LVSI), IA2, IB1, and IIA1 disease without any radiologic lymph node metastases [13]. Patients underwent SLNBx and were randomized to no additional surgery or systematic pelvic lymphadenectomy. Among patients with negative sentinel lymph nodes on the intraoperative frozen section (PHENIX I), a total of 420 patients had SLNBx alone while 418 had systematic lymphadenectomy. Patients who had SLNBx alone had shorter surgeries, with lower blood loss and perioperative complications. Following a median follow-up of 52 months, there was no difference in 3-year recurrence-free survival between the SLNBx and systematic LND groups (96.8% vs. 94.5%, HR 0.61, 95% CI: 0.33, 1.14). Interestingly, there were nine retroperitoneal nodal recurrences observed in the systematic lymphadenectomy group compared to none in the SLNBx alone group [13]. Limitations of the trial include the high adjuvant treatment use (even in the absence of lymph node metastases), use of methylene blue (instead of indocyanine green), as well as the lack of mandatory ultrastaging that could potentially detect micrometastases. A third trial (SENTICOL-III) is currently enrolling patients with early-stage disease and randomizes them to SLNBx alone or SLNBx with systematic lymphadenectomy. The co-primary endpoints are 3-year disease-free survival and quality of life with a target accrual of 950 patients and anticipated completion during 2026 [20]. The oncologic safety of SLNBx in the management of patients with apparent early-stage cervical cancer has also been evaluated in smaller studies [46]. A recent systematic review of the literature and meta-analysis identified six studies (two prospective and four retrospective) with a total of 992 patients and reported a cumulative 5-year disease-free and overall survival of 98% and 94%, respectively [46]. In an analysis of the National Cancer Database that included 15,711 patients with early-stage cervical cancer, 10.9% underwent SLNBx with an increasing utilization. Higher rates were observed among patients with smaller tumors, with 12.8% and 9% for those with ≤2 cm and 2–4 cm compared to 6.9% for >4 cm. While rates of lymph node metastases were comparable between patients who had SLNBx or systematic lymphadenectomy, the latter had a lower likelihood of prolonged hospitalization and comparable overall survival [47].

## 5. Fertility-Sparing Management in Cervical Cancer

Cervical cancer is diagnosed in approximately 45% of patients during their reproductive years, placing fertility preservation at the forefront of treatment decisions [48,49,50]. While extrafascial hysterectomy remains the gold standard for FIGO 2018 Stage IA1–IB1 disease confined to the cervix, fertility-sparing management (FSM) has transitioned from an experimental concept to a mainstream oncologic strategy for appropriately selected patients highly desirous of conserving their childbearing capacity, offering recurrence-free survival rates exceeding 95% and live birth rates between 55 and 85% in those attempting conception [25,48,49,51]. FSM is reserved for patients with early-stage disease, specifically FIGO 2018 stages IA1 with lymphovascular space invasion (LVSI), IA2, and IB [48,49,52]. Key eligibility criteria include tumor size ≤2 cm, negative pelvic lymph nodes, and clear endocervical margins on diagnostic conization. The presence of deep stromal invasion or substantial LVSI generally precludes conservative management due to their association with parametrial involvement and higher recurrence risk [51,52,53]. Regarding histology, FSM is acceptable in squamous cell carcinoma (SCC), adenocarcinoma (AC), and adenosquamous carcinoma (ASC). More aggressive histologies, including neuroendocrine and clear cell carcinomas, are generally excluded due to their poor prognosis and high recurrence rates [53]. Accurate preoperative tumor extent assessment using pelvic MRI and examination under anesthesia (EUA) is essential for surgical planning and eligibility determination, as emphasized in institutional protocols and recent guidelines [51,54]. MRI and EUA ensure precise volume assessment and minimize undertreatment risk. The FERTISS multicenter study highlighted the importance of strict size criteria, reporting a threefold increase in recurrence among tumors >2 cm, regardless of surgical radicality [22,54].

The choice of procedure is individualized based on tumor characteristics, patient anatomy, and reproductive goals. Less radical procedures such as conization and simple trachelectomy are increasingly favored when oncologic safety is not compromised. This choice is dictated by the tumor size, depth of stromal invasion, and LVSI status [14,49,52,54]:

• Conization or simple trachelectomy is the preferred approach for tumors ≤ 2 cm, offering excellent reproductive outcomes with oncologic safety comparable to radical procedures.
• Vaginal radical trachelectomy (RT) is associated with the highest clinical pregnancy rates—up to 67.5%—and is typically used for tumors < 2 cm.
• Abdominal RT is reserved for tumors measuring 2–4 cm but carries lower pregnancy rates and increased obstetric morbidity [55,56,57,58,59,60].

For stages IA2 and higher, any FSM surgical technique should include sentinel lymph node biopsy (SLNB). SLNB has replaced full pelvic lymphadenectomy in many centers, supported by the SENTICOL and SENTIX/ENGOT-Cx2 trials for its diagnostic accuracy and reduced morbidity [51,57,61,62]

From a safety standpoint, clinical evidence supports conservative surgery for early-stage disease. The ConCerv trial showed that patients with IA2–IB1 tumors ≤ 2 cm managed with conization or simple trachelectomy and SLNB had a 5-year recurrence-free survival (RFS) of 96.7% and a 60% pregnancy rate [14]. Radical trachelectomy series report similar oncologic outcomes, with 5-year RFS > 95% and live birth rates of 55–70% for tumors ≤ 2 cm [58,60]. Systematic reviews confirm the low recurrence rates (3–5%) across conservative approaches, with no oncologic advantage to more radical procedures in tumors ≤ 2 cm [57,60,61].

Impact of Histology:

FSM has shown comparable outcomes across SCC, AC, and ASC when criteria are met. Recurrence rates range from 3 to 5% for SCC/AC and 3 to 7% for ASC, with live birth rates from 60 to 85% [25,50,60]. Rare histologies such as neuroendocrine and clear cell carcinoma are generally excluded. Limited case series suggest that FSM may be cautiously considered for select patients with early-stage clear cell tumors with strict individual counseling and intense long-term surveillance. Nonetheless, in the absence of robust, large-scale data, the current recommendations continue to advise against FSM for these histologies [25,63,64].

Age as a Determinant of FSM:

Perimenopausal FSM is rare and often yields poor reproductive outcomes. Although pregnancy after menopause is technically feasible using donor oocytes or previously cryopreserved oocytes/embryos, it is generally discouraged due to the increased obstetrical risks and underlying medical comorbidities [65]. Consequently, postmenopausal FSM is generally not pursued; instead, fertility planning centers on anticipatory oocyte/embryo cryopreservation and the use of gestational surrogacy [66].

FSM in Tumors > 2 cm: An Evolving Debate:

In tumors measuring 2–4 cm (FIGO IB2), FSM remains controversial. Some reports suggest that neoadjuvant chemotherapy (NACT) followed by conservative surgery may be feasible in carefully selected patients, with recurrence rates of 6–13% and pregnancy rates near 44%. However, the risk of preterm birth in this group is high—up to 44% [25,52,55]. The ongoing CONTESSA trial is investigating this approach, but until conclusive data are available, FSM in IB2 should be limited to clinical trials or highly selected patients with detailed counseling [21].

## 6. Surgical Staging for Locally Advanced Disease

In 2018, the FIGO staging for cervical cancer changed with the addition of using pathologic and radiographic findings to aid in staging. Currently, standard of care management for locally advanced disease in cervical cancer is a combination of cisplatin with external beam radiation followed by vaginal brachytherapy [51]. Furthermore, immunotherapy can be added to the treatment paradigm for patients with stage III and IV disease as a result of Keynote A18 [67]. Although cervical cancer diagnoses are on a decline in the United States, it is still a public health crisis worldwide. Approximately 37% of women diagnosed with cervical cancer are diagnosed with FIGO stage IB3–IVA disease [68]. Of those, 10–25% of these patients have positive para-aortic lymph nodes [69]. Multiple imaging modalities are used to stage cervical cancer including CT, MRI, and PET–CT. For lymph node assessment specifically, PET–CT has been shown to be superior with a high positive predicative value of 79.3% and high specificity of 97.7%, which was demonstrated in ACRIN6671/GOG0233 [70]. However, the debate continues as to whether to surgically stage these patients.

The decision to stage surgically as opposed to radiographically or with pathologic assessment in locally advanced cervical cancer remains controversial in the field of gynecologic oncology. In a meta-analysis that included five studies by Delara et al., there was no statistically significant difference in oncologic outcomes for patients with stage IB2–IVA cervical cancer between patients who were surgically staged and those who were clinically staged [71]. Another study, which was retrospective and included 647 patients from 10 French cancer centers over a 20-year period (1996–2016) [72], sought to assess the oncologic outcomes for patients treated with chemoradiation and negative imaging for para-aortic lymph nodes. A total of 57% of those patients had a PET–CT to evaluate their lymph nodes and 58% (377) patients underwent surgical staging, which included the removal of lymph nodes using the iliac bifurcation as their most distal border and the left renal vein as their most proximal. This study found that after controlling for confounders, patients who were surgically staged had improved disease-free survival (HR 0.64, 95% CI 0.46, 0.89) and overall survival (HR 0.43, CI: 0.27, 0.68), with similar recurrence patterns. A NCDB study including patients diagnosed with locally advanced disease from 2010 to 2015, which included 3540 patients, demonstrated that a low proportion of patients (9.4%) were surgically staged, with a decreasing trend over the study time period [73]. This study found that there was no difference in overall survival between patients that were staged surgically and patients that were staged clinically, after controlling for confounders. Interestingly, for patients who underwent surgical staging and had positive para-aortic lymph nodes, they had an overall worse survival (HR 2.09, 95% CI: 1.41, 3.12). However, it is important to note that these findings are from retrospective studies.

Given the limitations of retrospective studies, there have been multiple randomized trials (RCTs) to assess if surgical staging can provide superior outcomes for locally advanced cervical cancer. The first team to look at this question was Lai et al., who conducted an RCT for patients with FIGO stage IIB (≥4 cm), III, and IVA [74]. Patients had either a CT or MRI as their preoperative imaging. For the staging arm, lymphadenectomy was performed with the common iliac bifurcation as the distal border and the inferior mesenteric artery as the proximal border. For patients with positive para-aortic lymph nodes, they were treated with extended-field radiation without cisplatin as concurrent chemoradiation was the standard of care treatment. At the interim analysis, patients who underwent surgical staging had a worse progression-free survival (HR 3.13, 95% CI: 1.42, 6.89). This finding led to the trial closing early. Another trial, LiLACS, a Phase III international trial, sought to assess if pre-therapeutic laparoscopic staging followed by tailored chemoradiation improved overall survival compared to PET–CT staging followed by chemoradiation in FIGO Stages IB2 to IVA in patients with FDG avid pelvic nodes but negative para-aortic lymph nodes [15]. Unfortunately, this study was closed due to poor accrual. At this time, UTERUS-11, the Phase III trial conducted at multiple sites in Europe, is the best trial to date to provide robust evidence answering the question of whether to surgically stage in locally advanced cervical cancer [16]. This trial assessed the role of pre-treatment surgical staging in FIGO 2009 stage IIB–IVA between 2009 and 2013. Preoperative imaging consisted of abdominal MRI or CT. The most proximal border for the para-aortic lymph node dissection was the infrarenal area. It is important to note that PET–CT was not required and was performed in only 18% (47/255) of patients. For patients who were surgically staged, 51% had positive pelvic lymph nodes while 24% had positive para-aortic lymph nodes. Not surprisingly, more patients were upstaged in the surgical staging arm (33 vs. 8%). The study did not demonstrate a difference in disease-free survival between the groups (aHR 0.73, 95% CI: 0.40, 0.93). An ad hoc analysis by stage did show that there was a benefit to surgically staging in patients with stage IIB. An important criticism to consider regarding this trial is that PET–CT was not required to assess the status of lymph nodes. Furthermore, the recurrence patterns were similar for both arms. Thus, should we still continue to assess if there is an oncologic benefit to surgically staging patients with locally advanced cervical cancer? This question continues to be studied with continued research in this area. We look forward to the results of the ongoing PAROLA trial which is assessing if chemoradiation with tailored external beam radiation after surgically staging and pathologic evaluation of the para-aortic lymph nodes is associated with an improved disease-free survival in patients staged with PET/CT only [23].

## 7. Pelvic Exenteration

Pelvic exenteration (PE) is a rare, highly complex, radical surgical procedure for pelvic malignancies. It was first performed by Dr. Alexander Brunschwig, who pioneered this radical procedure. In gynecologic cancers, it serves as potential surgical option for patients with cervical, endometrial, vaginal, and vulvar cancer, with recurrent or persistent cervical cancer being the most frequent indication [75]. Given the high morbidity of this procedure, the ultimate goal of a PE is to obtain clear margins around the tumor with curative intent [76,77]. Pelvic exenteration can be further divided into anterior PE (includes a cystectomy and urethrectomy), posterior PE (rectosigmoid resection), and a total PE (all pelvic organs). Furthermore, PE can be classified as supralevator (preserving the endopelvic fascia and pelvic floor) or infralevator (resection of the levator ani muscles). Most importantly, having a multidisciplinary team and selecting the right patient for the procedure are key. Counseling patients for what to expect is critical as their surgery is life-changing, affecting patients physically and psychologically as well as their sexual function. Further, studies have shown a mortality rate of 5%, further stressing the importance of patient selection [78].

Because a PE is a complex procedure with a high morbidity rate, an RCT would not be feasible nor ethical. However, there are retrospective case series looking at PE in terms of surgical approach, feasibility, and oncologic outcomes. Li et al. published a case series of 38 patients with recurrent or persistent cervical cancer who underwent a PE [79]. The majority of patients underwent a total PE (52.6%). Early complications, defined as a complication within 30 days of surgery, occurred in 21 patients, while late complications, defined as after 30 days, occurred in 15 patients. Although there was a very small number of patients, this series demonstrated a median OS of 28.5 months and DFS of 23 months. A larger retrospective study using NCDB data looked at survival in 313 women with cervical cancer who underwent a pelvic exenteration [80]. Covariates included age at diagnosis, lymph node status, income, insurance status, stage, exenteration type, surgical margin status, and adjuvant treatment (radiation or chemotherapy). This study showed that median OS was greater in women with node-negative disease with a median survival of 73.2 months compared to 17.8 months in women with node-positive disease. As discussed earlier, this study mentioned the importance of patient selection given the high morbidity rate.

Emerging contemporary data further refine our understanding of patient selection and outcomes. A recent multi-institutional analysis reported that laterally extended endopelvic resections and compartment-based approaches may improve margin status and resectability in select recurrent cases, and highlighted the prognostic significance of complete R0 resection and the absence of pelvic sidewall involvement [81]. Moreover, updated contemporary perioperative outcomes from high-volume centers show that advances in reconstructive techniques—including myocutaneous flaps, continent urinary diversions, and enhanced recovery pathways—have contributed to reductions in major morbidity and improved postoperative functional recovery [81]. In parallel, ongoing prospective efforts to standardize selection criteria are underway, exemplified by the actively recruiting MIPEX (Minimally Invasive Pelvic Exenteration in Vaginal or Cervical Cancer Recurrence) clinical trial NCT06867445, which is evaluating structured preoperative assessment, functional optimization, and patient-reported outcomes in individuals undergoing PE for recurrent or persistent cervical cancer.

PEs are associated with both significant medical and surgical complications. Important medical complications include thromboembolic and acute renal events [82]. Key surgical complications include wound breakdown (superficial, dehiscence and evisceration, and necrotizing fasciitis), urinary diversion complications, and bowel-related events including postoperative ileus, bowel obstruction, anastomotic leaks, fistulas, and stomal complications. The surgical approach for pelvic exenteration is generally through a large laparotomy incision. This is to ensure the ample exposure of all pelvic and abdominal organs. This also allows exposure and space if there is a plan for intraoperative radiotherapy. The use of a robotic approach has been reported in the literature [83,84,85]. This approach can be considered based on the amount of disease in the pelvis and surgical expertise.

Emerging data suggest that a higher surgical volume is associated with superior outcomes. A recent population-based study that identified 1912 patients undergoing pelvic exenteration for gynecologic malignancies in 181 centers in the United States reported lower perioperative mortality among higher volume centers [24]. In another single institution study, higher surgeon experience was associated with decreased blood loss and requirements for intraoperative transfusion and a shorter hospital length of stay [86,87].

## 8. Conclusions

Surgical management of patients with cervical cancer has rapidly evolved over the past decade with an emphasis on less radical approaches that preserve quality of life without compromising oncologic outcomes. Further research is required to evaluate whether minimally invasive techniques with protective maneuvers can be employed.

## Data Availability

No data was created or analyzed. We performed a literature review utilizing the Cochrane, Embase, and PubMed databases.
